# Presence of genes for type III secretion system 2 in *Vibrio mimicus *strains

**DOI:** 10.1186/1471-2180-10-302

**Published:** 2010-11-29

**Authors:** Natsumi Okada, Shigeaki Matsuda, Junko Matsuyama, Kwon-Sam Park, Calvin de los Reyes, Kazuhiro Kogure, Takeshi Honda, Tetsuya Iida

**Affiliations:** 1 Laboratory of Genomic Research on Pathogenic Bacteria, International Research Center for Infectious Diseases, Research Institute for Microbial Diseases, Osaka University, Osaka, Japan; 2 Department of Bacterial Infections, Research Institute for Microbial Diseases, Osaka University, Osaka, Japan; 3 Pathogenic Microbes Repository Unit, International Research Center for Infectious Diseases, Research Institute for Microbial Diseases, Osaka University, Osaka, Japan; 4 Department of Food Science and Technology, College of Ocean Science and Technology, Kunsan National University, Kunsan, Korea; 5 Marine Ecosystem Dynamics, Atmosphere and Ocean Research Institute, the University of Tokyo, Tokyo, Japan

## Abstract

**Background:**

Vibrios, which include more than 100 species, are ubiquitous in marine and estuarine environments, and several of them e.g. *Vibrio cholerae*, *V. parahaemolyticus*, *V. vulnificus *and *V. mimicus*, are pathogens for humans. Pathogenic *V. parahaemolyticus *strains possess two sets of genes for type III secretion system (T3SS), T3SS1 and T3SS2. The latter are critical for virulence of the organism and be classified into two distinct phylogroups, T3SS2α and T3SS2β, which are reportedly also found in pathogenic *V. cholerae *non-O1/non-O139 serogroup strains. However, whether T3SS2-related genes are present in other *Vibrio *species remains unclear.

**Results:**

We therefore examined the distribution of the genes for T3SS2 in vibrios other than *V. parahaemolyticus *by using a PCR assay targeting both T3SS2α and T3SS2β genes. Among the 32 *Vibrio *species tested in our study, several T3SS2-related genes were detected in three species, *V. cholerae*, *V. mimicus *and *V. hollisae*, and most of the essential genes for type III secretion were present in T3SS2-positive *V. cholerae *and *V. mimicus *strains. Moreover, both *V. mimicus *strains possessing T3SS2α and T3SS2β were identified. The gene organization of the T3SS2 gene clusters in *V. mimicus *strains was fundamentally similar to that of *V. parahaemolyticus *and *V. cholerae *in both T3SS2α- and T3SS2β-possessing strains.

**Conclusions:**

This study is the first reported evidence of the presence of T3SS2 gene clusters in *V. mimicus *strains. This finding thus provides a new insight into the pathogenicity of the *V. mimicus *species.

## Background

The type III secretion system (T3SS) is possessed by gram-negative bacteria, especially those occurring in animal and plant pathogens, e.g. *Yersinia, Shigella, Salmonella, Pseudomonas *and *Escherichia *species [1-3]. The T3SS secretes and translocates effector proteins into the cytosol of eukaryotic cells, thus contributing to bacterial virulence against the host [1]. While the T3SS apparatus is well conserved in these bacteria, the specific properties of the effectors which are secreted via T3SS and symptomatic effects caused by the effectors on the host organism vary widely [1].

Vibrios are gram-negative γ-proteobacteria which are ubiquitous in marine and estuarine environments [4,5]. Several of the more than 100 *Vibrio *species are pathogens for fish, shellfish, coral, and mammals [6], and *Vibrio parahaemolyticus *was the first species in which the presence of T3SS was reported [7].

*V. parahaemolyticus *is a cause of food-borne gastroenteritis in humans, and almost all strains isolated from diarrheal patients produce the thermostable direct hemolysin (TDH) and/or the TDH-related hemolysin (TRH), which are encoded by the *tdh *and *trh *genes, respectively [8-10]. *V. parahaemolyticus *strains, which exhibit the Kanagawa phenomenon (KP), a beta-hemolysis detectable on a special blood agar (Wagatsuma agar) [11], possess two *tdh *genes, *tdhA *and *tdhS*, but not the *trh *gene [10,12,13]. In contrast, KP-negative clinical *V. parahaemolyticus *strains possess the *trh *gene only or both the *trh *and *tdh *genes. Genome sequencing of the KP-positive *V. parahaemolyticus *strain RIMD2210633 demonstrated that it possesses two sets of the genes for T3SS on chromosomes 1 and 2 (T3SS1 and T3SS2, respectively) [7]. It has further been demonstrated that T3SS2 is involved in enterotoxicity of the organism, and is considered to be an important factor in the pathogenicity of diarrheal illness [14]. The T3SS2 genes are located on a pathogenicity island (PAI) known as Vp-PAI on chromosome 2, and the genes were found in KP-positive, but not in KP-negative strains [7,14,15].

After the discovery of the T3SS genes in *V. parahaemolyticus*, other vibrios such as *V. alginolyticus*, *V. harveyi*, *V. tubiashii *and *V. cholerae *were also found to possess the genes for T3SS [14,16-18]. While the T3SSs of *V. alginolyticus*, *V. harveyi *and *V. tubiashii*, are more closely related to T3SS1 of *V. parahaemolyticus *[14], that of *V. cholerae *is similar to T3SS2 of *V. parahaemolyticus *[17]. In addition, several studies have demonstrated that some *V. cholerae *non-O1/non-O139 serogroup strains, which do not possess the cholera toxin gene, do possess a set of T3SS genes in a PAI (VPI-2) on their chromosome [17,19]. It has further been suggested that the T3SS of non-O1/non-O139 *V. cholerae *is also involved in the pathogenicity of the bacterium [17].

In our most recently reported study, we used the sequencing and PCR assay of the genomic DNA of the TH3996 strain to detect the presence of a novel PAI (Vp-PAI_TH3996_) in *trh*-positive (KP-negative) *V. parahaemolyticus *strains [20]. The Vp-PAI_TH3996 _was found to contain a set of genes for T3SS, and the T3SS of the TH3996 strain to be essential for the enterotoxicity of this strain [20]. Phylogenetic analysis indicated that the T3SS genes of TH3996 are related to that of RIMD2210633, but belong to distinct lineage, with the former known as T3SS2β and the latter as T3SS2α [20]. Subsequent studies showed that T3SS2α and T3SS2β are present in, respectively, KP-positive and *trh*-positive *V. parahaemolyticus *strains and are also distributed among pathogenic *V. cholerae *non-O1/non-O139 serogroup strains [20].

A previous study examined the distribution of the T3SS2-related genes in *Vibrio *species, but tested only for the presence of the T3SS2α genes and in a limited number of strains from each species [14]. In this study, we re-investigated the distribution of the genes for T3SS2 in various *Vibrio *species and targeted both the T3SS2α and T3SS2β genes.

## Results

### Distribution of the T3SS2-related genes in *Vibrio *species

To analyze the distribution of the T3SS2-related genes in *Vibrio *species other than *V. parahaemolyticus*, PCR assays were performed using oligonucleotide primer pairs (see Additional file 1) which target the T3SS2-related genes present in the Vp-PAI, i.e., *vscN2 *(encodes the ATPase), *vscC2N2R2S2T2U2*, *vcrD2 *(apparatus proteins of T3SS), *vopB2D2 *(translocons), or *vopCLP *(effectors) [14,21-24], for 32 *Vibrio *species. The design of the PCR primer pairs was based on the gene sequences in strains RIMD2210633 or TH3996, representing T3SS2α or T3SS2β, respectively (see Additional file 1). We tested multiple strains of several species in the genus *Vibrio *which are implicated as pathogenic for humans, that is, *V. vulnificus *(10 strains), *V. fluvialis *(12 strains), *V. furnissii *(12 strains), *V. hollisae *(5 strains), *V. cholerae *(46 strains) and *V. mimicus *(15 strains). For other species, one strain of each was tested (see Additional file 2).

The assay demonstrated that, in addition to part of the *V. cholerae *strains, as previously reported, amplicons of the expected size of at least several of the T3SS2 genes were obtained from all of the *V. hollisae *strains and some of the *V. mimicus *strains. However, none of the genes tested in any of the remaining 29 species could be amplified (see Additional file 2).

Among the 46 non-O1/non-O139 *V. cholerae *strains isolated from patients (28 strains) or environments (18 strains), we obtained the amplicons of at least one gene encoding the apparatus protein of the T3SS2α genes from 10 strains (see below). In two *V. cholerae *strains, which constitute the PCR products of T3SS2β genes, at least six genes for the apparatus and two genes for the translocons could be amplified (see Additional file 2). We therefore concluded that the aforementioned 10 *V. cholerae *strains were T3SS2α-positive and the two were T3SS2β-positive. Of these 12 T3SS2-positive strains, only one, the *V. cholerae *strain RIMD2214415, which possesses T3SS2α genes, was isolated from the environment. Therefore, as far as we could determine in this study, T3SS2 genes of *V. cholerae *tend to be found in clinical strains rather than in environmental isolates.

In all of the five *V. hollisae *strains tested, the amplicons for three genes of T3SS2α, *vscN2, vscR2 and vscT2*, were obtained with the PCR assay, but no other T3SS2α genes or any T3SS2β genes could be amplified. The PCR products for *vscN2R2T2 *could be partially sequenced, which confirmed that the amplicons that could be obtained are more closely related to the T3SS2α than to the T3SS2β genes (data not shown).

The PCR products of the genes for T3SS2 were detected in nine of 15 clinical or environmental *V. mimicus *strains. The genes encoding the apparatus proteins of T3SS2, *vscN2C2R2T2U2 *and *vcrD2*, were amplified by PCR in all the T3SS2-positive *V. mimicus *strains, although the amplicons for the genes encoding effector proteins, i.e., *vopCLP*, could not be obtained in a few of these strains (see Additional file 2). Of the nine T3SS2-positive strains, at least six genes for the apparatus proteins and two genes for the translocons of T3SS2α genes could be amplified from eight strains, while PCR amplification led to the detection in a *V. mimicus *strain of the amplicons of the T3SS2β genes, i.e., six genes encoding the apparatus proteins *vscN2C2R2T2U2 *and *vcrD2*, two genes encoding the translocons *vopB2D2*, and two genes for the regulators *vtrAB*. In the other six *V. mimicus *strains, no amplicons of the genes for either type of T3SS2 could be obtained (see Additional file 2). Of the nine T3SS2-positive *V. mimicus *strains, eight were therefore identified as T3SS2α-positive, and one as T3SS2β-positive. These findings suggest that, in addition to their distribution in *V. parahaemolyticus *and *V. cholerae *strains, the genes for T3SS2 are found in *V. hollisae *and *V. mimicus *strains, although only three T3SS2 genes were detected in *V. hollisae *strains.

### Distribution of *tdh *or *trh *in T3SS2-positive *V. cholerae *and *V. mimicus *strains

In *V. parahaemolyticus*, the strains which possess the T3SS2 gene cluster also possess the *tdh *and/or *trh *genes [7,20]. To examine whether the *tdh *or *trh *genes also coexist in T3SS2-possessing *V. cholerae *and *V. mimicus *strains, we performed a PCR assay using the pair of primers (see Additional file 1) which target the *tdh *or *trh *genes of *V. parahaemolyticus *RIMD2210633 or TH3996 strains, respectively. Among the T3SS2-possessing *V. mimicus *strains, the *tdh *gene could be detected in all T3SS2α-positive strains, although no amplicons of the *trh *gene could be obtained in *V. mimicus *strains, while neither of the amplicons could be obtained in the T3SS2-positive *V. cholerae *strains. To analyze the distribution of the *tdh *or *trh *genes in 12 T3SS2-positive *V. cholerae *strains, we performed an additional PCR assay using the primer set (see Additional file 1) targeting the region between A33_1702 and the downstream region of the *V. cholerae *AM-19226 strain which is homologous with *tdh *of *V. parahaemolyticus*. PCR products could be obtained for both the T3SS2α- and T3SS2β-positive *V. cholerae *strains, except for the RIMD2214321 (T3SS2α-possessing) strain (data not shown). These results suggest that the *tdh *gene may be related to the presence of the T3SS2 gene cluster in *V. cholerae *and *V. mimicus *strains.

### Gene organization of the T3SS2 gene cluster in *V. mimicus*

The results presented here demonstrated that some *V. mimicus *strains possess the genes for T3SS2α or T3SS2β. Since the gene organizations of the T3SS2 gene cluster in the organism were completely unknown, we attempted to analyze the gene organization of the T3SS2 region in *V. mimicus *strains. To this end, we performed PCR scanning against the genomic DNA of T3SS2-positive *V. mimicus *strains RIMD2218080 (T3SS2α) and RIMD2218067 (T3SS2β) by using six PCR primer pairs for each strain (see Additional file 3).

In the T3SS2α-positive strain RIMD2218080, PCR products of the expected size were detected for all primer pairs (data not shown), thus suggesting that the gene organization of the T3SS2 gene cluster in strain RIMD2218080 is similar to that of the T3SS2α gene cluster in *V. parahaemolyticus *strain RIMD2210633. In the T3SS2β-positive strain RIMD2218067, amplicons of the expected size were obtained for five primer pairs, although the size of the product obtained for a primer pair between the *vopD2 *and *vopC *genes was notably larger, by approximately 5 kb, than that of the region of the *V. parahaemolyticus *TH3996 (data not shown). However, the PCR product which was amplified between *vopD2 *and *vopP *was the same size as that of the *V. parahaemolyticus *TH3996 strain. This suggested that the gene organization of T3SS2 of *V. mimicus *RIMD2218067 is similar to that of T3SS2β of *V. parahaemolyticus*, while a genetic element of approximately 5 kb, which could not be detected in the region of *V. parahaemolyticus *strain TH3996, may exist in the region between the *vopP *and *vopC *genes in *V. mimicus *strain RIMD2218067.

These findings suggest that the gene organization of the T3SS2 gene clusters, both T3SS2α and T3SS2β, in *V. mimicus *strains are basically similar to those of the *V. parahaemolyticus *and *V. cholerae *strains.

### Phylogenetic analysis of the T3SS2-related genes in *V. mimicus*

Next, we analyzed the phylogeny of the T3SS2 genes identified in *V. mimicus *strains. The purified amplicons of the genes for *vscN2R2T2 *in the T3SS2-positive *V. mimicus *strains were sequenced and the nucleotide sequences thus obtained were used for phylogenetic analysis. In addition, we used the nucleotide sequences of the three T3SS2 genes of the two *V. parahaemolyticus *strains RIMD2210633 and TH3996, and the four *V. cholerae *strains, AM-19226, 1587 and 623-39, as well as V51, identified to date. Phylogenetic trees for each of the genes were constructed with the Neighbor-Joining (NJ) method.

**Figure 1:**
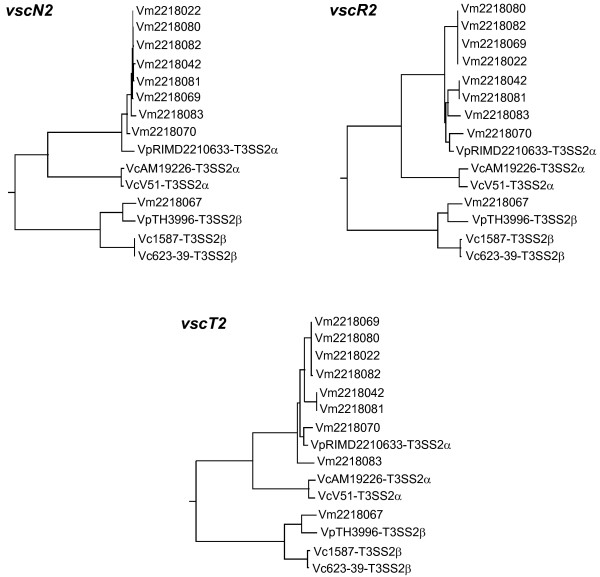
**Phylogenetic analysis of the T3SS2 genes**. Phylogenetic trees of the three T3SS2 genes (*vscN2R2T2*) constructed with the NJ method. Abbreviations of the 15 strains used for the analysis: VpTH3996-T3SS2β: *V. parahaemolyticus *str. TH3996; VpRIMD2210633-T3SS2α: *V. parahaemolyticus *str. RIMD2210633; VcAM19226-T3SS2α: *V. cholerae *str. AM-19226; Vc1587-T3SS2β: *V. cholerae *str. 1587; Vc623-39-T3SS2β: *V. cholerae *str. 623-39; VcV51-T3SS2: *V. cholerae *str. V51; Vm2218022: *V. mimicus *str. RIMD2218022; Vm2218042: *V. mimicus *str. RIMD2218042; Vm2218067: *V. mimicus *str. RIMD2218067; Vm2218069: *V. mimicus *str. RIMD2218069; Vm2218070: *V. mimicus *str. RIMD2218070; Vm2218080: *V. mimicus *str. RIMD2218080; Vm2218081: *V. mimicus *str. RIMD2218081; Vm2218082: *V. mimicus *str. RIMD2218082; Vm2218083: *V. mimicus *str. RIMD2218083. Sequence information was obtained from the NCBI. The computer program CLUSTAL W was used for the amino acid sequence alignment and phylogenetic analysis.

The analysis demonstrated that the PCR products of the T3SS2 genes in *V. mimicus *strains RIMD2218022, 2218042, 2218069, 2218070, 2218080, 2218081, 2218082 and 2218083 belong to the cluster containing the T3SS2α genes of *V. parahaemolyticus *strain RIMD2210633 and that of *V. cholerae *strains AM-19226 and V51 (Figure 1). In contrast, the amplicons obtained from the T3SS2 genes in the *V. mimicus *strain RIMD2218067 were found to be closely related to the T3SS2β genes in the *V. parahaemolyticus *TH3996 strain and *V. cholerae *strains 1587 and 623-39 (Figure 1). These findings confirmed that, similar to the findings for *V. parahaemolyticus *and *V. cholerae *strains, the T3SS2 of *V. mimicus *strains could be classified into two phylogroups, T3SS2α and T3SS2β.

### Presence and absence of the genes in VPI-2 and Vp-PAI

**Figure 2:**
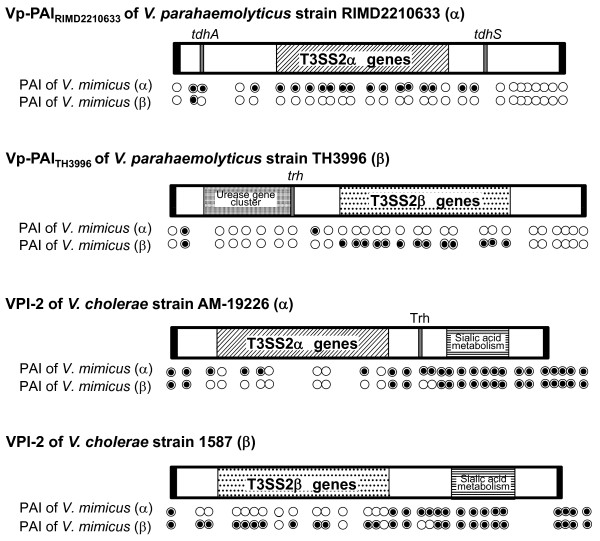
**Comparison of the structure of PAI in *V. parahaemolyticus, V. cholerae and V. mimicus***. Schematic representation of the structure of the PAI in *V. parahaemolyticus *RIMD2210633 (containing T3SS2α) and TH3996 (containing T3SS2β) strains and in *V. cholerae *AM-19226 (containing T3SS2α) and 1587 (containing T3SS2β) strains. Names of the various *V. parahaemolyticus *and *V. cholerae *strains are shown along the left side. Black boxes represent core chromosomal genes flanking the PAI region in *V. parahaemolyticus *or *V. cholerae *strains. Horizontally striped boxes represent sialic acid metabolism regions, and the checkered box represents the urease gene cluster, while diagonally striped and dotted boxes represent T3SS2 regions, and white boxes other ORFs in PAI regions. White circles represent the ORFs which were tested for the presence or absence of ORFs in *V. mimicus *strains, and black circles indicate the presence of such ORFs.

Both the T3SS2 gene cluster of *V. parahaemolyticus *and the T3SS gene cluster of *V. cholerae *can be found on PAIs [7,19,20]. In *V. cholerae *non-O1/non-O139 strains AM-19226, NRT36S, V51, 1587 and 623-39, the PAI (VPI-2) contains the genes for sialic acid metabolism in addition to the genes for T3SS (Figure 2) [19]. In *V. parahaemolyticus *strains RIMD2210633 and TH3996, on the other hand, the homologues for sialic acid metabolism were not found in the Vp-PAI (Figure 2). The gene compositions of the PAI cassettes in *V. parahaemolyticus *and *V. cholerae*, except for the T3SS gene cluster, were thus clearly distinct. To compare the gene organization of the PAI of *V. mimicus *with that of the PAIs of *V. parahaemolyticus *and *V. cholerae*, we used additional PCR assays to determine the presence or absence of open reading frames (ORFs), which occur only in the Vp-PAI of *V. parahaemolyticus *or the VPI-2 of *V. cholerae*, in T3SS2-positive *V. mimicus *strains. The ORFs on the PAIs of *V. parahaemolyticus *and *V. cholerae *strains, except for the T3SS2 genes, could be amplified with the primer sets that were designed by using the ORF sequences on the Vp-PAI in *V. parahaemolyticus *strains RIMD2210633 and TH3996 and those on VPI-2 in *V. cholerae *strains AM-19226 and 1587 as templates against the genomic DNA of nine *V. mimicus *T3SS2-positive strains (see Additional file 4, 5, 6, 7). Some of the ORFs on the Vp-PAI of *V. parahaemolyticus *strains, could be amplified in the *V. mimicus *strains tested, but most could not (see Additional file 4, 5, 6, 7, Figure 2). In contrast, most of the non-T3SS ORFs on VPI-2 of *V. cholerae *could be amplified in the T3SS-positive, but not in the T3SS2-negative *V. mimicus *strains (data not shown).

These findings suggest that the composition of the *V. mimicus *PAIs containing the T3SS genes, if present, may be more closely related to that of *V. cholerae *VPI-2 than of *V. parahaemolyticus *Vp-PAI (Figure 2).

### Cytotoxicity assay of mutant strains

Previous studies have demonstrated that T3SS2s of *V. parahaemolyticus *RIMD2210633 and TH3996 as well as *V. cholerae *AM-19226 contribute to the pathogenicity of these organisms [14,17,20,22-24]. To determine the possible contribution of T3SS2 to the pathogenicity of *V. mimicus *strains, we compared the cytotoxicity of the wild-type and mutant strains for cultured cell lines. T3SS-deficient mutants were constructed by disruption of the homologue of the *vscN2 *gene, which encodes an ATPase of T3SS2, in *V. mimicus *RIMD2218042 (α type) and RIMD2218067 (β type) strains. To confirm the deletion of the *vscN2 *gene, PCR amplification using oligonucleotide primer pairs was performed (see Additional file 1 and 8). The growth of the mutant strains in LB medium (1% NaCl) was indistinguishable from that of the parental strains (data not shown). Both *V. mimicus *RIMD2218042 and RIMD2218067 strains were cytotoxic for Caco-2 cells at 3 h post-infection. The cytotoxicity of both the T3SS2α- and T3SS2β-deficient mutant strains tended to decrease, but there were no significant differences between T3SS2α- and T3SS2β-deficient mutant strains and their parental strains (see Additional file 9).

## Discussion

A recent study of ours demonstrated that two lines of distinct lineage of the T3SS2 gene cluster, T3SS2α and T3SS2β, are present in the KP-positive and *trh*-positive *V. parahaemolyticus *strains, respectively [20]. Although a previously reported study using dot blot analysis could not detect the genes for T3SS2 in 16 *Vibrio *species, the probes and PCR primers used in previous studies were designed based on the sequence information of the T3SS2α genes in *V. parahaemolyticus *strain RIMD2210633 [7,14]. Since the T3SS2β genes cannot be detected by either PCR amplifications or comparative genomic hybridization analysis targeting the T3SS2α genes [7,15], we re-investigated the distribution of the T3SS2 genes, both T3SS2α and T3SS2β, in *Vibrio *species.

To examine the distribution of the genes for T3SS2 in vibrios other than *V. parahaemolyticus*, we performed a PCR assay using PCR primer pairs targeting both the T3SS2α and T3SS2β genes. Of the 32 *Vibrio *species tested, the T3SS2-related genes were detected in three species, *V. cholerae*, which was previously reported, as well as *V. hollisae *and *V. mimicus*.

In *V. hollisae *strains, only three genes for T3SS2α, *vscN2, vscR2, and vscT2*, were detected. Nevertheless, the fact that the PCR reactions for these three genes were positive in all the five *V. hollisae *strains tested is intriguing. We speculate that the other genes for T3SS2α might be absent in these particular *V. hollisae *strains, or that the sequences of the other genes included variations that would make PCR amplification with the primer pairs used in this assay difficult. These possibilities should be examined in the future by more detailed genetic analyses, e.g. sequencing of the region flanking the T3SS2-related genes.

**Figure 3:**
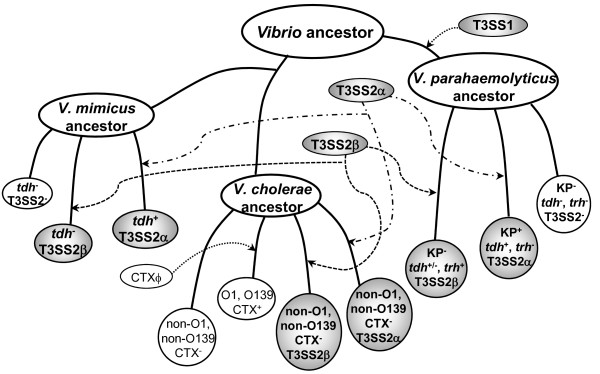
**Schematic representation of the hypothetical evolutionary acquisition of the T3SS-related gene cluster in *V. parahaemolyticus*, *V. cholerae *and *V. mimicus***. Lineage is based on the presence of each of the determinants, for example, *tdh*, *trh*, CTX and T3SS2. The shaded ellipses show the T3SS-related gene clusters, bold lines represent the evolutionary process, and circles represent the strains of *V. parahaemolyticus*, *V. cholerae *and *V. mimicus*, while shaded circles indicate that the strains possess T3SSα or T3SSβ. Broken lines indicate that the T3SS gene clusters or CTX have been acquired by horizontal gene transfer while the organisms were evolving.

A previous study showed that the T3SS2-related genes are present in *V. mimicus *strains [25]. In our study, the PCR assay also demonstrated the presence of the T3SS2 genes in *V. mimicus *strains. Of the 15 *V. mimicus *strains tested in this study, 12 strains were isolated from patients and three from the environment, with all T3SS2-positive strains isolated from patients. There were eight T3SS2α-positive and one T3SS2β-positive strain among the T3SS2-positive *V. mimicus *strains. The gene organization of the T3SS2 gene cluster in the *V. mimicus *strains containing T3SS2α or T3SS2β, was basically similar to that of the *V. parahaemolyticus *and *V. cholerae *strains. Ours is thus the first study to demonstrate that the two distinct types of T3SS2 gene clusters, T3SS2α and T3SS2β, are present not only in *V. parahaemolyticus *and *V. cholerae *but also in *V. mimicus *strains. Furthermore, we could show that the structures of the *V. mimicus *PAIs containing the T3SS genes may be more closely related to those of *V. cholerae *VPI-2 than of *V. parahaemolyticus *Vp-PAI (Figure 2). In contrast, the ORFs in VPI-2 were not detected in any of the T3SS-negative *V. mimicus *strains. This implies, therefore, that the similar PAI cassettes containing the T3SS2 gene cluster were acquired through horizontal gene transfer in *V. cholerae *and *V. mimicus *(Figure 3).

The PCR primer pairs used in this study were found to be useful for detecting as well as distinguishing the genes for T3SS2α and T3SS2β in *Vibrio *species. In particular, the PCR assays targeting the three genes, *vscN2, vscR2 and vscT2*, produced stable and reliable results for detection of T3SS2-related genes. We therefore consider that, for determining the presence or absence of these genes, PCR amplification using the primer pairs for the *vscN2R2T2 *genes of T3SS2α or T3SS2β is effective and rapid. Although only a limited number of strains of the non-human pathogenic *Vibrio *species was examined in this study, more extensive studies of those species using more strains may well reveal the presence of the T3SS2 genes in vibrios other than the ones reported here.

Previous studies showed that the T3SSs of *V. parahaemolyticus *and *V. cholerae *contribute to their pathogenicity for humans [14,17,20,22-24]. In *V. mimicus*, a bacterium which is known to be a causative agent of gastroenteritis in humans, the hemolysin was previously reported as a major virulence factor [26]. To assess the function of T3SS of *V. mimicus *in pathogenicity in our study, we evaluated the cytotoxicity of *V. mimicus *for Caco-2 cells because *V. parahaemolyticus *had a T3SS2-dependent cytotoxic effect on Caco-2 cells [22]. The results indicated that both T3SS2α-possessing and T3SS2β-possessing *V. mimicus *strains showed the cytotoxic activity on Caco-2 cells in this assay. Although we could not detect statistically significant differences between T3SS-deficient mutants and parental strains, there was a tendency for the cytotoxicity of T3SS-deficient mutants to diminish than that of the parental mutants. A previous report showed that the deletion of the hemolysin gene in *V. mimicus *significantly reduced fluid accumulation in rabbit ileal loop tests, but the mutant partially retained this action, which suggests that, besides the hemolysin, *V. mimicus *may contain an additional virulence determinant(s) [26]. It is therefore possible that T3SS is a candidate for the previously unidentified virulence determinant in pathogenic *V. mimicus *strains for humans. The observed ambiguous differences in cytotoxicity between the mutants and the parental strains may be due to insufficient expression of T3SS of *V. mimicus *under the culturing conditions used in this study, because it is still unclear what the optimal conditions are for inducing T3SS of *V. mimicus*. This possibility needs to be examined in future studies.

## Conclusions

This study demonstrated the presence of the gene cluster for T3SS2α or T3SS2β in *V. mimicus*, a bacterium which is known to be a causative agent of gastroenteritis in humans. Since it was reported that the T3SSs of *V. parahaemolyticus *and *V. cholerae *contribute to their pathogenicity for humans, the T3SS in *V. mimicus *identified in this study also might be a candidate virulence factor of this organism for humans. This possibility needs to be examined in future studies.

## Methods

### Bacterial strains and growth conditions

All the *Vibrio *species strains were obtained from the Pathogenic Microbes Repository Unit, International Research Center for Infectious Diseases, Research Institute for Microbial Diseases, Osaka University. The culture temperatures were 15°C for *V. logei *and *V. salmonicida *and 10°C for *V. wondanis*, while all other bacteria were cultured at 25°C. The bacteria were grown with shaking in Luria-Bertani (LB) broth (tryptone, 1%; yeast extract, 0.5%) with 3% NaCl for *V. parahemolyticus *and in Difco marine broth 2216 for *V. nigripulchritudo*, *V. pectenicida *and *V. halioticoli*. Other bacteria were grown in LB broth with 1% NaCl.

### Oligonucleotide primers and PCR conditions

Additional file 1 shows the oligonucleotide primers used in this study. Chromosomal DNA from *Vibrio *species strains was extracted for PCR as previously described [20]. For detection of the presence of the T3SS2 genes in related *Vibrio *species, PCR using the EX-PCR Kit (Takara Shuzo, Kyoto, Japan) was performed. The PCR conditions were as follows: after initial denaturation at 94°C for 3 min, a cycle of 94°C for 30 s, 55°C for 30 s, and 72°C for 30 s, 45 s, 1 min or 1.5 min was repeated 30 times. PCR scanning of the *V. mimicus *was performed using genomic DNA as a template and with a long accurate (LA) PCR kit (Takara Shuzo) as previously described [20]. Custom-synthesized oligonucleotides for the PCR were purchased from GeneDesign (Osaka, Japan).

### DNA sequencing and informatic analysis

To sequence the DNA fragments amplified by PCR, the fragments were purified with the PCR Gel Extraction Kit (QIAGEN, Valencia, CA) according to the manufacturer's protocol. DNA sequencing was performed with the ABI PRISM 3130 (Applied Biosystems, Foster City, CA) and the BigDye v3.1 cycle sequencing kit (Applied Biosystems). The Genetyx sequence analysis program (Software Development, Tokyo, Japan) was used for computer analysis of DNA sequences. Homology searches against deposited sequences were performed with the aid of data from the National Center for Biotechnology Information (NCBI) using the BLAST network service http://www.ncbi.nlm.nih.gov and the BLAST service at the Genome Information Research Center http://genome.naist.jp/bacteria/vpara/. Sequence information was obtained from the NCBI. The computer program CLUSTAL W was used for the nucleotide sequence alignment and phylogenetic analysis.

### Construction of *vscN2 *deletion mutant strains of *V. mimicus*

A four-primer PCR technique was used to engineer an in-frame deletion mutation as described previously [14]. Briefly, the upstream and downstream sequences of *vscN2 *of T3SS2α or T3SS2β were amplified using the pairs listed in Additional file 1. The two fragments, amplified with primers 1 and 3, and 2 and 4, respectively, were used as templates for a second PCR using primers 1 and 4, which generated a PCR product containing the desired deletion. The amplified fragments were then sequenced and subcloned into an R6K-*ori *suicide vector pYAK1 and transformed into *E. coli *SM10λ*pir*.

### Cytotoxicity assays

For cytotoxicity assays, eukaryotic cells were seeded at 3 × 10^4 ^cells well^-1 ^in 96-well plates and cultured for 48 h to confluency. The cells were co-cultured with PBS-washed bacteria at a multiplicity of infection (moi) of 10 for 2- 6 h. The release of lactate dehydrogenase (LDH) into the medium was quantified by using a CytoTox96 non-radioactive cytotoxicity kit (Promega) according to the manufacturer's instructions. The LDH release (per cent cytotoxicity) was calculated with the following equation: [optical density at 492 nm (OD_492_) of experimental release - OD_492 _of spontaneous release]/(OD_492 _of maximum release - OD_492 _of spontaneous release) × 100. Spontaneous release is defined as the amount of LDH released from the cytoplasm of uninfected cells, and maximum release as the total amount of LDH released after the complete lysis of uninfected cells.

### Statistical analysis

Statistical significance was determined with the *t *test. A *P *value of < 0.05 was considered statistically significant.

### Nucleotide sequence accession numbers

The nucleotide sequence data reported in this paper will appear in the DDBJ, EMBL, and GenBank nucleotide sequence database under accession numbers AB560976, AB560977, AB560978, AB560979, AB560980, AB560981, AB5609782, AB560983, AB560984, AB560985, AB560986, AB560987, AB560988, AB560989, AB560990, AB560991, AB560992, AB560993, AB560994, AB560995, AB560996, AB560997, AB560998, AB560999, AB561000, AB561001, and AB561002.

## Authors' contributions

NO designed the study, performed most experiments, interpreted the data and drafted the manuscript. SM shared a part of PCR work, sequencing and cytotoxicity assay, helped to design the study and draft the manuscript. JM and KK isolated and collected *Vibrio *strains used in the work. KSP and CR assisted study design and data interpretation. TH and TI coordinated the work and drafted the manuscript. All authors read and approved the manuscript.

## Supplementary Material

Additional file 1 **Table S1. The primer sequences used in this study**. Oligonucleotide primers sequences used in this study are listed in this table. Click here for file

Additional file 2 **Table S2. Distribution of the T3SS2α or T3SS2β genes on Vp-PAI in 32 *Vibrio *species**. The species and strain ID of *Vibrio *strains used in this study are listed in this table. Click here for file

Additional file 3 **Figure S1. Gene organization of the T3SS2α and T3SS2β gene clusters in *V. parahaemolyticus *strains**. Genetic organization of T3SS2 in *V. parahaemolyticus *TH3996 (β type) and RIMD2210633 (α type) strains. Genes are indicated by arrows, with red arrows indicating the genes encoding putative apparatus proteins of T3SS2, blue arrows the genes encoding putative regulatory and effector proteins of T3SS2, and gray arrows the genes encoding hypothetical proteins. The colors of the arrows are identical to those used in a previous report of ours [20]. The 12 lines with arrowheads at both ends, representing PCR-Vpa1-Vpa6 and PCR-Vpb1-Vpb6, designate the regions that were amplified for PCR scanning. Click here for file

Additional file 4 **Table S3. Distribution of the ORFs on PAI in *V. parahaemolyticus*, *V. cholerae *and *V. mimicus *strains**. The species, strain ID, serogroup, source and year of isolation of *V. parahaemolyticus*, *V. cholerae *and *V. mimicus *strains are listed in this table. A, gene encoding the putative apparatus protein of T3SS; T, gene encoding the putative translocon of T3SS; R, gene encoding the putative regulatory protein of T3SS; E, gene encoding the putative effector protein of T3SS; nt, not tested. The numbered columns correspond to ORFs in *V. parahaemolyticus *RIMD2210633 strain; 1, VPA1309; 2, VPA1312; 3, VPA1314 (*tdh *gene); 4, VPA1373; 5, VPA1376; 6, VPA1380; 7, VPA1387; 8, VPA1388; 9, VPA1393; 10, VPA1394; 11, VPA1395; 12, VPA1396; 13, VPA1397. Click here for file

Additional file 5 **Table S4. Distribution of the ORFs on PAI in *V. parahaemolyticus*, *V. cholerae *and *V. mimicus *strains**. The species, strain ID, serogroup, source and year of isolation of *V. parahaemolyticus*, *V. cholerae *and *V. mimicus *strains are listed in this table. A, gene encoding the putative apparatus protein of T3SS; T, gene encoding the putative translocon of T3SS; R, gene encoding the putative regulatory protein of T3SS; E, gene encoding the putative effector protein of T3SS; nt, not tested. The numbered columns correspond to ORFs in *V. parahaemolyticus *TH3996 strain; 1, orf-1; 2, RPI08 (*ureG*); 3, RPI15 (*nikA*); 4, RPI17 (*nikC*); 5, RPI20 (*ureR*); 6, RPI23 (*ospB*); 7, RPI27; 8, RPI80; 9, RPI82; 10, RPI85; 11, RPI87; 12, RPI89; 13, RPI92; 14, orf + 1. Click here for file

Additional file 6 **Table S5. Distribution of the ORFs on PAI in *V. parahaemolyticus*, *V. cholerae *and *V. mimicus *strains**. The species, strain ID, serogroup, source and year of isolation of *V. parahaemolyticus*, *V. cholerae *and *V. mimicus *strains are listed in this table. A, gene encoding the putative apparatus protein of T3SS; T, gene encoding the putative translocon of T3SS; R, gene encoding the putative regulatory protein of T3SS; E, gene encoding the putative effector protein of T3SS; nt, not tested. The numbered columns correspond to ORFs in *V. cholerae *AM-19226 strain; 1, A33_1654; 2, A33_1655; 3, A33_1657; 4, A33_1659; 5, A33_1661; 6, A33_1663; 7, A33_1697; 8, A33_1700; 9, A33_1703; 10, A33_1704; 11, A33_1706; 12, A33_1713; 13, A33_1715; 14, A33_1719; 15, A33_1722; 16, A33_1724; 17, A33_1726; 18, A33_1728. Click here for file

Additional file 7 **Table S6. Distribution of the ORFs on PAI in *V. parahaemolyticus*, *V. cholerae *and *V. mimicus *strains**. The species, strain ID, serogroup, source and year of isolation of *V. parahaemolyticus*, *V. cholerae *and *V. mimicus *strains are listed in this table. A, gene encoding the putative apparatus protein of T3SS; T, gene encoding the putative translocon of T3SS; R, gene encoding the putative regulatory protein of T3SS; E, gene encoding the putative effector protein of T3SS; nt, not tested. The numbered columns correspond to ORFs in *V. cholerae *1587 strain; 1, A55_1978; 2, A55_1980; 3, A55_1981; 4, A55_1982; 5, A55_1983; 6, A55_1984; 7, A55_1988; 8, A55_1989; 9, A55_B0297; 10, A55_B0300; 11, A55_2005; 12, A55_2008; 13, A55_2011; 14, A55_2013; 15, A55_2016; 16, A55_2018; 17, A55_2021; 18, A55_2023; 19, A55_2027; 20, A55_2030; 21, A55_2031. Click here for file

Additional file 8 **Figure S2. PCR amplification of the *vscN2 *deletion mutant *V. mimicus *strains**. Parental strains (ca. 1200 bp), T3SS-deficient mutant strains (ca. 600bp). The size of the products of the mutant strains was notably smaller, by approximately 600 bp, than that of parental strains, including that the mutant strains of *vscN2 *genes of *V. mimicus *were constructed. 1, *V. mimicus *RIMD2218042 (T3SS2α-possessing) strain; 2, *V. mimicus *RIMD2218042Δ*vscN2 *(T3SS2α-deficient mutant) strain; 3, *V. mimicus *RIMD2218067 (T3SS2β-possessing) strain; 4, *V. mimicus *RIMD2218067Δ*vscN2 *(T3SS2β-deficient mutant) strain. Click here for file

Additional file 9 **Figure S3. Cytotoxicity induced by *V. mimicus *against Caco-2 cells**. Caco-2 cells were infected with bacteria at an moi of 10. After infection, cytotoxicity was assayed by measuring total cellular LDH release into the cellular supernatant. The amount of LDH released by Caco-2 cells was measured 3 h after infection with RIMD2218042 (T3SS2α-possessing) or RIMD2218042Δ*vscN2 *(T3SS2α-deficient mutant) or RIMD2218067 (T3SS2β-possessing) or RIMD2218067Δ*vscN2 *(T3SS2β-deficient mutant) strains. Click here for file
