# Supplementary material for: Presence of genes for type III secretion system 2 in Vibrio mimicus strains
Source: BMC Microbiol. 2010 Nov 29;10:302. doi: 10.1186/1471-2180-10-302 (PMC3004890; doi:10.1186/1471-2180-10-302)

T3SS2β gene cluster of *V. parahaemolyticus* TH3996 strain

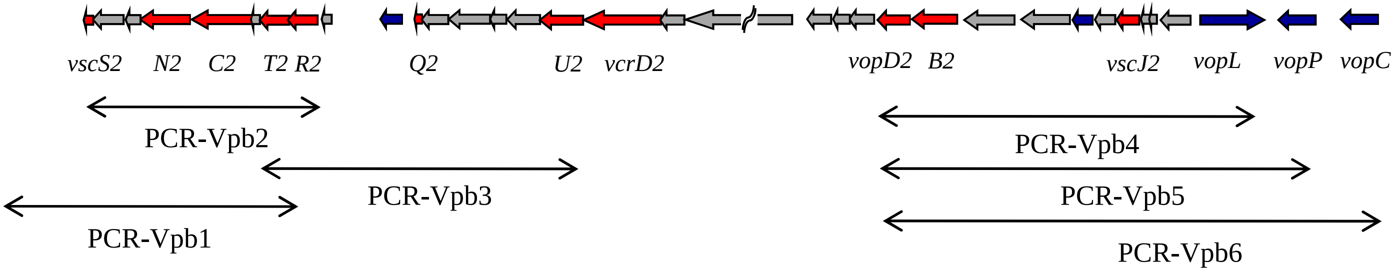

T3SS2α gene cluster of *V. parahaemolyticus* RIMD2210633 strain

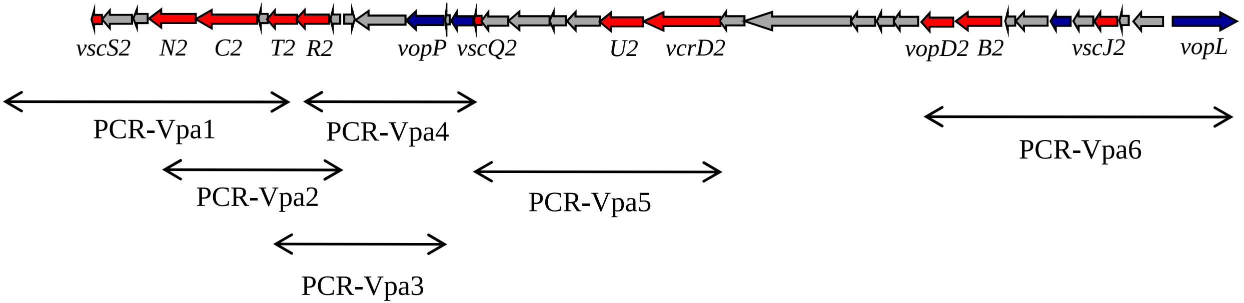

Supplement: Additional file 3 — Figure S1. Gene organization of the T3SS2α and T3SS2β gene clusters in V. parahaemolyticus strains. Genetic organization of T3SS2 in V. parahaemolyticus TH3996 (β type) and RIMD2210633 (α type) strains. Genes are indicated by arrows, with red arrows indicating the genes encoding putative apparatus proteins of T3SS2, blue arrows the genes encoding putative regulatory and effector proteins of T3SS2, and gray arrows the genes encoding hypothetical proteins. The colors of the arrows are identical to those used in a previous report of ours [20]. The 12 lines with arrowheads at both ends, representing PCR-Vpa1-Vpa6 and PCR-Vpb1-Vpb6, designate the regions that were amplified for PCR scanning. [file 1471-2180-10-302-S3.PDF]
