# Supplementary figures and images for: Presence of genes for type III secretion system 2 in Vibrio mimicus strains
Source: BMC Microbiol. 2010 Nov 29;10:302. doi: 10.1186/1471-2180-10-302 (PMC3004890; doi:10.1186/1471-2180-10-302)

M

1

2

M

3

4

(bp)

1500

1000

500

100

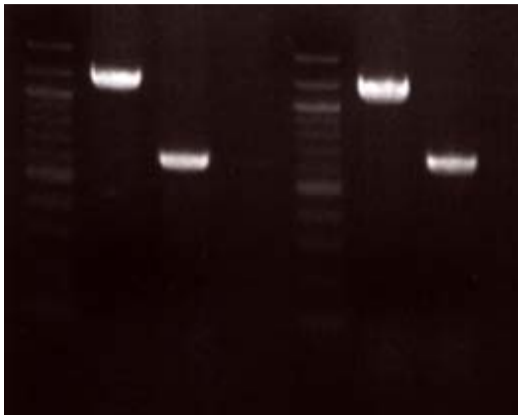

Supplement: Additional file 8 — Figure S2. PCR amplification of the vscN2 deletion mutant V. mimicus strains. Parental strains (ca. 1200 bp), T3SS-deficient mutant strains (ca. 600bp). The size of the products of the mutant strains was notably smaller, by approximately 600 bp, than that of parental strains, including that the mutant strains of vscN2 genes of V. mimicus were constructed. 1, V. mimicus RIMD2218042 (T3SS2α-possessing) strain; 2, V. mimicus RIMD2218042ΔvscN2 (T3SS2α-deficient mutant) strain; 3, V. mimicus RIMD2218067 (T3SS2β-possessing) strain; 4, V. mimicus RIMD2218067ΔvscN2 (T3SS2β-deficient mutant) strain. [file 1471-2180-10-302-S8.PDF]

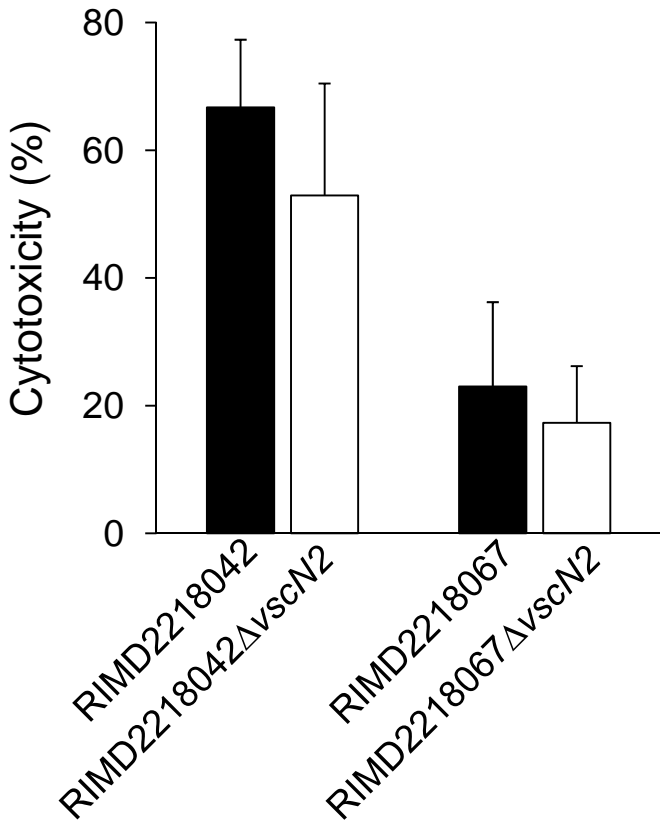

Supplement: Additional file 9 — Figure S3. Cytotoxicity induced by V. mimicus against Caco-2 cells. Caco-2 cells were infected with bacteria at an moi of 10. After infection, cytotoxicity was assayed by measuring total cellular LDH release into the cellular supernatant. The amount of LDH released by Caco-2 cells was measured 3 h after infection with RIMD2218042 (T3SS2α-possessing) or RIMD2218042ΔvscN2 (T3SS2α-deficient mutant) or RIMD2218067 (T3SS2β-possessing) or RIMD2218067ΔvscN2 (T3SS2β-deficient mutant) strains. [file 1471-2180-10-302-S9.PDF]
